# New-Onset Psoriatic Arthritis Following COVID-19 mRNA Vaccination in a Psoriatic Patient Under Anti-tumor Necrosis Factor Alpha Biologic Treatment: What Now?

**DOI:** 10.7759/cureus.50723

**Published:** 2023-12-18

**Authors:** Marisa Sousa, Sofia Gersão, Hugo B Sousa

**Affiliations:** 1 Unidade de Saúde Familiar Samora Correia (Primary Care Unit), Agrupamento de Centros de Saúde Estuário do Tejo (Administração Regional de Saúde de Lisboa e Vale do Tejo) National Health Service, Samora Correia, PRT; 2 Unidade de Cuidados de Saúde Personalizados Águeda V (Primary Care Unit), Agrupamento de Centros de Saúde Baixo Vouga (Administração Regional de Saúde do Centro) National Health Service, Águeda, PRT

**Keywords:** autoimmune disease flare, biologic treatment, mrna vaccine, covid-19 vaccination, reactive arthritis, psoriasis, psoriatic arthritis

## Abstract

During the COVID-19 pandemic, anti-SARS-CoV-2 vaccines were quickly developed and administered to the population worldwide. As is expected with new vaccine products, adverse reactions following immunization have been reported, namely, the development and/or exacerbation of autoimmune/autoinflammatory diseases, including rheumatic diseases. Here, we report a clinical case of a 56-year-old woman with a 44-year history of moderate-to-severe plaque psoriasis under treatment with an anti-tumor necrosis factor alpha biosimilar (adalimumab) with good control of skin disease and without rheumatic involvement to date who came to us with complaints of migratory polyarthralgia starting one week after receiving the second dose of the BNT162b2 COVID-19 mRNA vaccine. The condition progressed over the following months and a diagnosis of psoriatic arthritis was established. Biologic treatment was switched to an anti-interleukin 17A (secukinumab), with a very good clinical cutaneous and articular response, which was sustained up to the present moment.

The mechanisms behind the exacerbation or new-onset of autoimmune/autoinflammatory diseases after receiving anti-COVID-19 vaccines are not yet fully understood, requiring further investigation. It is also not known whether rheumatic symptoms post-COVID-19 infection will have similar mechanisms to rheumatic symptoms post-anti-COVID-19 vaccination. With the continuing worldwide vaccination against SARS-CoV-2, clinicians need to be prepared to discuss the risks and benefits of vaccination and should be aware that it may cause or exacerbate immune disorders such as psoriatic arthritis, warranting close follow-up in terms of disease progression and treatment.

## Introduction

For the past three years, following the COVID-19 pandemic, anti-SARS-CoV-2 vaccines were quickly developed and have been administered to the population worldwide to prevent serious illness and reduce morbidity and mortality. The most common adverse reactions to COVID-19 mRNA vaccination are general disorders and administration-site conditions [1], such as local injection-site reactions and flu-like symptoms. Nonetheless, other adverse effects of such vaccines have been reported, namely, the development of autoimmunity/inflammatory phenomena, such as thrombotic events, thrombocytopenia and myocarditis, and the exacerbation of underlying autoimmune/inflammatory diseases, such as psoriasis and rheumatological diseases, including psoriatic arthritis (PsA), rheumatoid arthritis, systemic lupus erythematosus, polymyalgia rheumatica, and vasculitis [2-4]. The appearance of reactive arthritis secondary to vaccination or SARS-CoV-2 infection has also been reported [5,6].

According to the European Alliance of Associations for Rheumatology (2021) and the American College of Rheumatology (2021), anti-COVID-19 vaccines are tolerable and safe in patients with autoimmune/inflammatory rheumatological diseases, with a low percentage of severe exacerbations (0.6% in 4.4% of total exacerbations) and severe adverse effects (0.5% in 37% of total adverse effects) [7,8]. In a recent systematic review aimed at studying new-onset rheumatic immune-mediated inflammatory diseases following COVID-19 vaccinations, a total of 271 cases from 39 countries were reported from January 2021 to May 2023, with 50% of patients developing rheumatic disease shortly after the second dose of the vaccine [3]. Vasculitis was the most common clinical presentation (31.7%), followed by connective tissue diseases (24.3%) and inflammatory arthritis (20.3%) [3]. In addition, recent systematic reviews have also evidenced both new-onset psoriasis and psoriasis flares as cutaneous adverse events following COVID-19 vaccination [9,10].

Here, we report a clinical case of a 56-year-old woman with a 44-year history of moderate-to-severe plaque psoriasis under treatment with an anti-tumor necrosis factor alpha (TNF-α) biosimilar (adalimumab 40 mg, subcutaneously, every two weeks) with good control of cutaneous manifestations, without prior rheumatic involvement, who exhibited complaints of migratory polyarthralgia starting one week after receiving the second dose of the BNT162b2 COVID-19 mRNA vaccine.

This article was previously presented as a meeting abstract and poster at the 14th Practical Course of Rheumatology in Primary Health Care organized by “Prismédica - Department of Medical Congresses” on September 22nd, 2022.

## Case presentation

**Figure 1:**
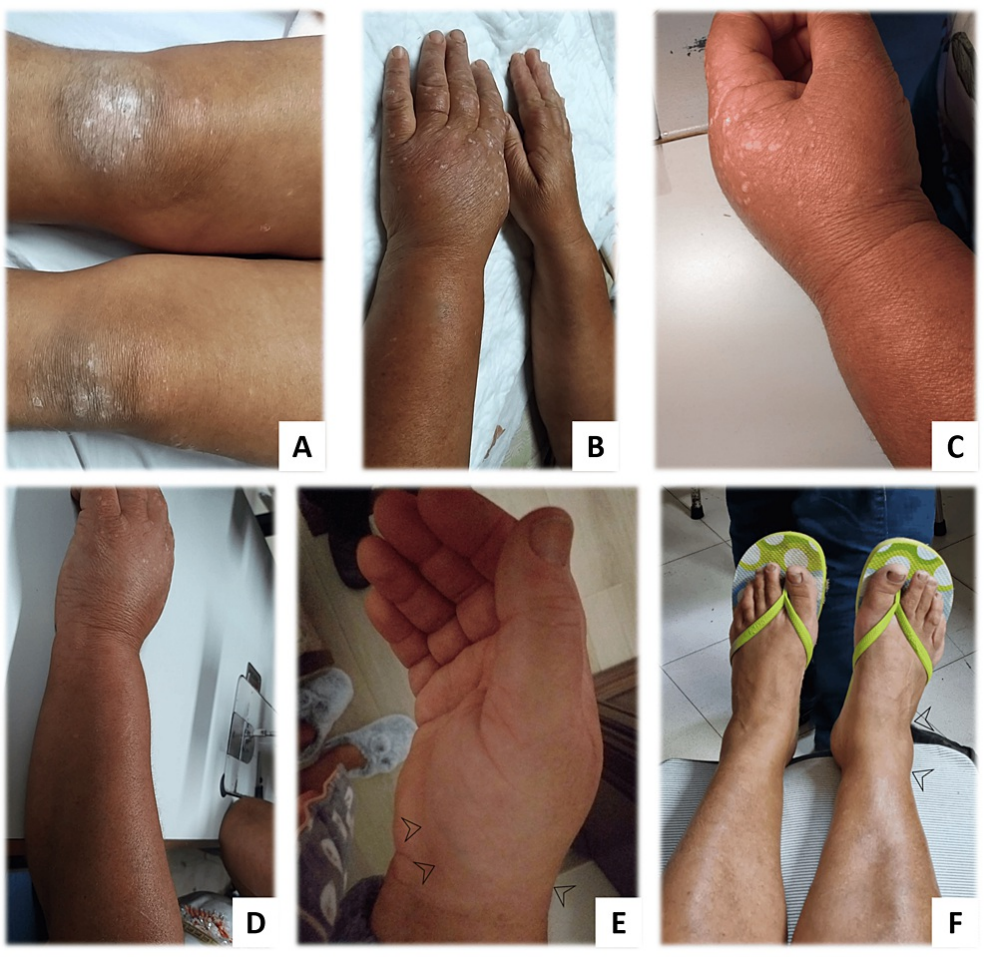
Initial migratory polyarthritis. Inflammatory signs of initial migratory polyarthralgia (August 2021): the first joint affected was the right knee (A), migrating to the left wrist (with forearm flexor tenosynovitis) (B, C, and D) and subsequently to the right wrist (E, black arrowheads showing soft tissue swelling) and right tibiotarsal joint (F, black arrowheads showing soft tissue swelling).

We report the clinical case of a 56-year-old woman, with a history of moderate-to-severe plaque psoriasis since the age of 12, without rheumatic symptoms to date, and controlled under biological therapy with anti-TNF-α biosimilar (adalimumab 40 mg, subcutaneously, every two weeks - Amgevita®) for the previous four months. She presented to our clinic in August 2021, one month after receiving the second dose of the BNT162b2 (mRNA, Pfizer-Biotech®) anti-COVID-19 vaccine, complaining of new migratory polyarthralgia during the previous three weeks. The patient reported inflammatory symptoms (redness, warmth, swelling, pain, and loss of function) at the level of the right knee joint that migrated to the left wrist (with flexor tenosynovitis in the forearm) and later to the right wrist and right Achilles tendon with ensuing enthesitis (Figure 1). She was suffering from severe debilitating pain of inflammatory rhythm (Visual Analogue Scale (VAS) of Pain 90/100; VAS of Activity 90/100); Clinical VAS of Activity 80/100), partially relieved by ibuprofen 600 mg (on demand). She experienced no ophthalmological symptoms, genitourinary or bowel movement changes, inflammatory bowel disease, sacroiliitis, inflammatory axial pain, fever, or other constitutional symptoms. At this point, we instituted a baseline symptomatic therapy consisting of naproxen 500 mg (twice daily) and omeprazole 20 mg (once daily) (while still on adalimumab).

**Figure 2:**
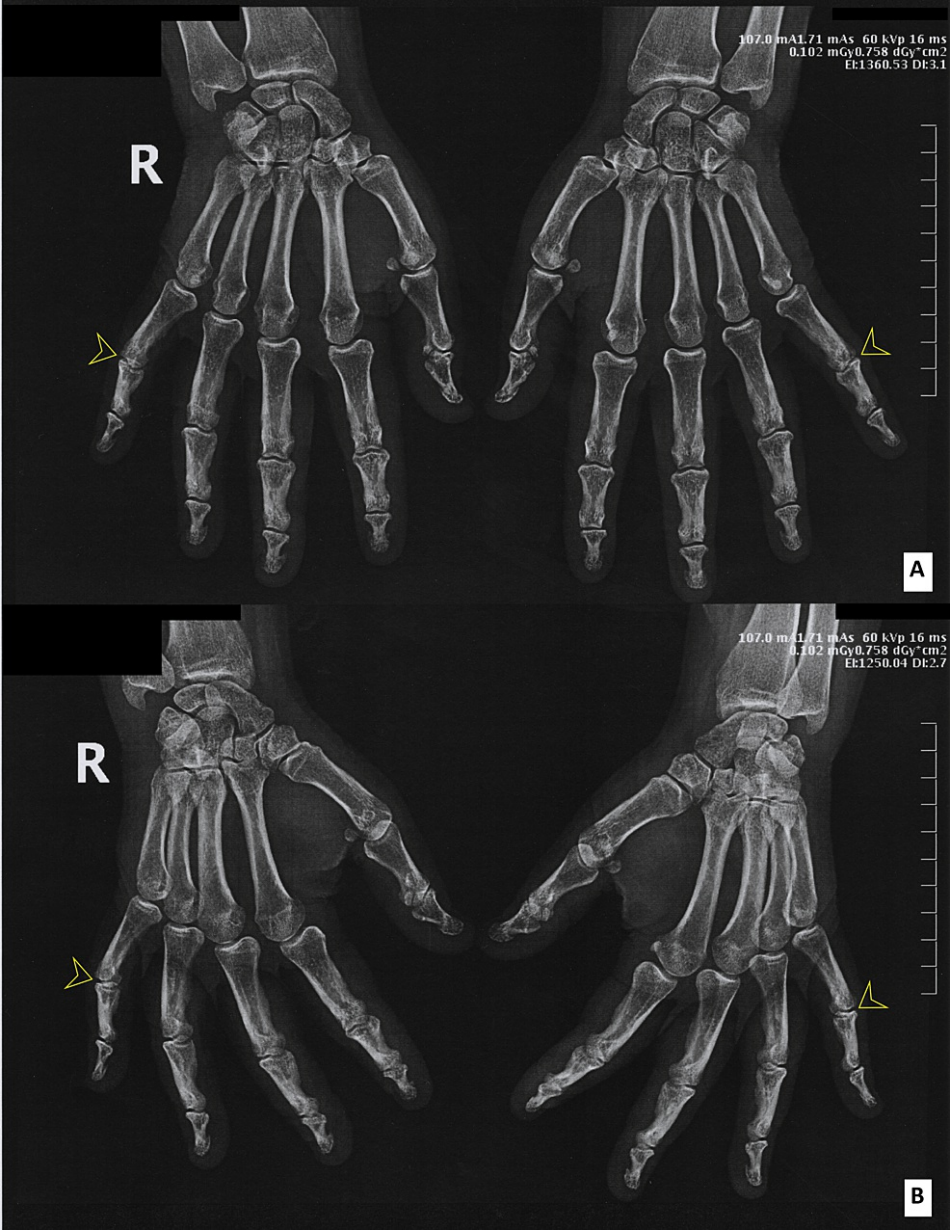
Osteoarthritis of the hands. X-ray of the hands (A: frontal view; B: oblique view): radiological evidence of bilateral incipient osteoarthritis of the proximal interphalangeal joint of the fifth finger (yellow arrowheads showing diminished intra-articular space).

The condition progressed over the following month (September 2021) to become an additive symmetric polyarthritis, which upon objective examination displayed bilateral involvement of the elbows, metacarpophalangeal joints, proximal interphalangeal joints (PIPs), tibiotarsal joints, and metatarsophalangeal joints, as well as of the left knee but not the right. The Disease Activity Score in 28 Joints (DAS-28) and Disease Activity Index for Psoriatic Arthritis (DAPSA) were 7.58 and 60, respectively, indicating high disease activity. At that time, bloodwork revealed the following: hemoglobin 13.3 g/dL (normal range: 12.0-16.0 g/dL); leukocytes 5,010/mm^3^ (normal range: 4,500-11,000/mm^3^); platelets 164,000/mm^3^ (normal range: 150,000-400,000/mm^3^); C-reactive protein (CRP) 21.8 mg/L (normal range: <3 mg/L); erythrocyte sedimentation rate (ESR) 40 mm/hour (normal range: <20 mm/hour); and lactate dehydrogenase (LDH) 341 U/L (normal range: 45-90 U/L); negative serologies for human immunodeficiency virus 1/2 (HIV), hepatitis B and C, and venereal disease research laboratory; antistreptolysin O 7 U/mL (normal range: <200 U/mL); anti-DNAseB 79 U/L (normal range: <200 U/L); negative anti-nuclear antibodies, anti-cyclic citrullinated peptide, rheumatoid factor, and human leukocyte antigen B27; aldolase 23.2 U/L (normal range: <7.6 U/L); creatine phosphokinase 82 U/L (normal range: 25-90 U/L); and normal protein electrophoresis. Radiological evidence of bilateral incipient osteoarthritis of the PIP of the fifth finger stood out, but no other significant bone or joint changes in the remainder of the affected joints were evident (Figure 2). Due to the progression of the rheumatic disease and difficulty in controlling pain, prednisolone 7.5 mg (daily) was initiated in mid-September. The patient was also referred to the Rheumatology consultation at the same hospital as her Dermatology follow-up.

During October 2021, the patient still had significant joint involvement with uncontrollable pain and cutaneous exacerbation of abdominal psoriasis. The bloodwork revealed increased inflammatory parameters: CRP of 54.5 mg/L and ESR of 55 mm/hour. Due to the patient’s previous intolerance to methotrexate, leflunomide 10 mg (once daily) was added to the prednisolone treatment (7.5 mg daily, started the month before). During November 2021, she still experienced a few exacerbations of the rheumatic disease with almost overlapping inflammatory parameters (CRP of 39.6 mg/L and ESR of 55 mm/hour); therefore, leflunomide was increased to 20 mg (once daily), which resulted in an improvement in the pain and joint inflammation and overall better control of the rheumatic disease.

By the end of November 2021, her rheumatic disease was more stable and controlled but the skin disease had worsened. She was evaluated at the Rheumatology and Dermatology hospital appointments in December 2021 and the clinical examination revealed extensive scaly erythematous plaques on the abdomen, trunk, arms, legs, and scalp (Psoriasis Area Severity Index (PASI) of 25.2) with little joint involvement (VAS Pain 40/100; VAS Activity 30/100; Clinical VAS Activity 20/100; and DAS28 and DAPSA scores of 4.36 and 28, respectively, indicating moderate disease activity). The diagnosis of PsA was then established.

A joint decision by the Rheumatology and Dermatology departments was made to switch to an anti-interleukin (IL)-17A biologic due to previous psoriasis therapeutic failure with etanercept and intolerance to infliximab (both anti-TNF-α therapies the patient had been submitted to for psoriasis treatment in previous years before starting on adalimumab). She was further prescribed calcium and vitamin D (in December 2021).

**Figure 3:**
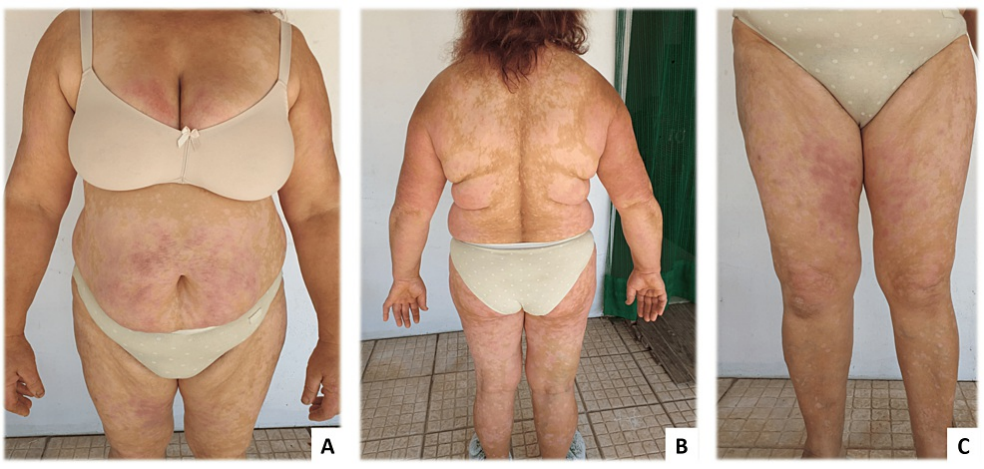
Therapeutic response to secukinumab. Skin and joint therapeutic response to secukinumab 300 mg, subcutaneously, monthly (anti-IL17-A, Cosentyx®) just two months after starting therapy in June 2021. There is nearly complete remission of the psoriatic skin lesions (the extension of lesions corresponded to what we can now see as healed hypopigmented skin) and inflammatory signs at the joint level are absent (A, B, and C).

In March 2022, the patient stopped adalimumab and started secukinumab 300 mg monthly by subcutaneous injection (anti-IL17A, Cosentyx®) with a good cutaneous and articular therapeutic response after two months (PASI 3.6, patient; VAS Pain 30/100; VAS Activity 30/100; Clinical VAS Activity 10/100; DAS28 2.64; and DAPSA 7, both scores showing low disease activity, close to remission) (Figure 3), and she was weaned off of her prednisolone and leflunomide.

In July 2022, the patient had COVID-19 with concurrent exacerbation of her polyarthralgia for two days, which resolved with a short cycle of prednisolone 10 mg. Since then and up to the submission of this case report (November 2023), the patient has been on monthly secukinumab administration (plus naproxen on demand) with good control of her cutaneous and rheumatic diseases.

## Discussion

Vaccination is intended to stimulate the immune system toward protective immunity, yet the precise mechanisms responsible for the exacerbation of autoimmune/autoinflammatory diseases after taking anti-COVID-19 vaccines have not yet been identified. Unlike previous vaccines, both mRNA and viral vector vaccines encode the SARS-CoV-2 spike (S) protein, which is known to be a potent inducer of type I interferon and proinflammatory cytokines, a pathway shared by immune-mediated inflammatory diseases. On the other hand, the S protein is also the major target for neutralizing antibodies arising from natural infection and monoclonal antibodies used for treatment [11].

Moreover, there is no clarification on whether the post-COVID-19 infection rheumatological symptoms and those after COVID-19 vaccination arise by similar pathophysiological mechanisms. One could argue that the mechanism for psoriasis exacerbation might probably be similar to that which occurs with other vaccines [12]. In such a case, the vaccine would stimulate the production of IL-6 post-vaccination, which then would stimulate T helper (Th)-17 cells to produce IL-22, inducing the proliferation of keratinocytes. Additionally, patients with polyarthralgias post-COVID-19 infection are known to have high levels of IL-6 in circulation (in addition to an increase in CRP and ESR), and these levels could be predictors of post-COVID-19 arthritis [13]. Finally, the cytokine hyperactivation that results from COVID-19 infection (with increases in IL-1, IL-6, and TNF-α) appears to be similar to that which occurs in RA [14].

In this clinical case, we have presented a patient with longstanding cutaneous psoriasis without a previous history of rheumatic disease and for whom the anti-COVID-19 vaccination may have triggered severe and debilitating PsA just one week after the second dose of the vaccine. It is noteworthy that this occurred while under biological therapy with an anti-TNF-α agent. Studies have shown that both cellular and humoral immunity elicited by the COVID-19 vaccines are impaired and a faster waning of humoral and cellular markers of immunity occurs in patients under treatment with anti-TNF-α agents [15,16]; therefore, we can hypothesize that other immune pathways may be involved, such as lipid nanoparticle triggered inflammation, molecular mimicry, bystander activation, or yet other factors [17,18]. The psoriatic flare in our case appeared to be associated with the administration of prednisolone, yet the effect of vaccination cannot be dismissed [9,10]. Disease remission was achieved and maintained through a switch from an anti-TNF-α to an anti-IL17-A therapy. Also worthy of note, the patient was already on her third anti-TNF-α agent for psoriasis treatment when she first exhibited symptoms of PsA. To date, reports from the literature support that the exacerbation or appearance of rheumatological diseases following COVID-19 vaccinations seem to have a good prognosis as they are still rare, short-lived, and respond well to steroids and other immunosuppressant agents [3,4,6,14].

## Conclusions

Further studies are required to unravel the pathophysiology driving the autoimmune and inflammatory phenomena behind new-onset presentation or exacerbation of previous rheumatological disease, be it either post-infection or post-vaccination against COVID-19, to determine which individuals are at greatest risk for such conditions. The incidence of new-onset rheumatic immune-mediated inflammatory diseases, treatments, and prognosis of these patients should be thoroughly investigated and its findings should be used to help patient management and create vaccines with a better adverse reaction profile.

Amid the ongoing worldwide vaccination against SARS-CoV-2, clinicians need to be prepared to discuss the risks and benefits of vaccination, acknowledging that it may exacerbate immune disorders and planning for a close follow-up in terms of disease progression and treatment.
